# Seroepidemiology of human *Toxoplasma gondii *infection in China

**DOI:** 10.1186/1471-2334-10-4

**Published:** 2010-01-07

**Authors:** Yue Xiao, Jigang Yin, Ning Jiang, Mei Xiang, Lili Hao, Huijun Lu, Hong Sang, Xianying Liu, Huiji Xu, Johan Ankarklev, Johan Lindh, Qijun Chen

**Affiliations:** 1 Key Laboratory of Zoonosis, Ministry of Education, Jilin University, Xi An Da Lu 5333, Changchun 130062, PR China; 2 The Second Hospital of Jilin University, Ziqiang Street 218, Changchun 10041, PR China; 3 Institute of Pathogen Biology, Chinese Academy of Medical Sciences, Beijing, Dong Dan San Tiao, Beijing 100730, PR China; 4 The Sixth Hospital of Changchun City, North Round Road 4596, Changchun 130040, PR China; 5 Changzheng Hospital, Shanghai, Fengyang Road 415, Shanghai 200003, PR China; 6 Department of Parasitology, Mycology and Environmental Microbiology, Swedish Institute for Infectious Disease Control, Nobels väg 18, 171 82 Solna, Sweden

## Abstract

**Background:**

Toxoplasmosis is an important zoonotic parasitic disease worldwide. In immune competent individuals, *Toxoplasma gondii *preferentially infects tissues of central nervous systems, which might be an adding factor of certain psychiatric disorders. Congenital transmission of *T. gondii *during pregnancy has been regarded as a risk factor for the health of newborn infants. While in immune-compromised individuals, the parasite can cause life-threatening infections. This study aims to investigate the prevalence of *T. gondii *infection among clinically healthy **i**ndividuals and patients with psychiatric disorders in China and to identify the potential risk factors related to the vulnerability of infection in the population.

**Methods:**

Serum samples from 2634 healthy individuals and 547 patients with certain psychiatric disorders in Changchun and Daqing in the northeast, and in Shanghai in the south of China were examined respectively for the levels of anti-*T. gondii *IgG by indirect ELISA and a direct agglutination assay. Prevalence of *T. gondii *infection in the Chinese population in respect of gender, age, residence and health status was systematically analyzed.

**Results:**

The overall anti-*T. gondii *IgG prevalence in the study population was 12.3%. In the clinically healthy population 12.5% was sero-positive and in the group with psychiatric disorders 11.3% of these patients were positive with anti-*T. gondii *IgG. A significant difference (P = 0.004) was found between male and female in the healthy population, the seroprevalence was 10.5% in men versus 14.3% in women. Furthermore, the difference of *T. gondii *infection rate between male and female in the 20-19 year's group was more obvious, with 6.4% in male population and 14.6% in female population.

**Conclusion:**

A significant higher prevalence of *T. gondii *infection was observed in female in the clinically healthy population. No correlation was found between *T. gondii *infection and psychiatric disorders in this study. Results suggest that women are more exposed to *T. gondii *infection than men in China. The data argue for deeper investigations for the potential risk factors that threat the female populations.

## Background

*T. gondii *is an intracellular parasite that can infect almost all mammals and the importance of this parasite in food safety, human health and animal husbandry has been well recognized. Although the parasites remain dormant in people with normal immune competence, they do pose threats to individuals who are immuno-compromized. Examples could be patients with AIDS or organ transplantation [1-3]. It has been estimated that up to one third of the world's population has been infected by *T. gondii *with endemicity from around 10% to 70% [2,4,5]. Further more, up to 14.8% of AIDS patients in Southeast Asia region were reported with toxoplasmosis in the central nervous systems [6]. Both primary and re-infection during pregnancy are high risk factors especially to the foetus [7,8]. Thus surveillance of *T. gondii *infection and the distribution of the oocysts in the living environments have been regarded as an important measure to prevent the disease.

Due to the fact that *T. gondii *mainly infects cells in the brain of immune competent individuals, it has been postulated that certain psychiatric disorders might be a link to the concurrent infection [9-11]. In several studies, patients with schizophrenia were found to have a higher tendency of *T. gondii *infection [12-14], but there has been no conclusive correlation between *T. gondii *infection and psychiatric disease [15].

In this study, we investigated the prevalence of anti-*T. gondii *IgG in the sera of more than 3000 Chinese individuals living in the southern and northern regions of China, among which, a panel of 547 serum samples were collected from patients with certain psychiatric disorders. It was found that the general infection rate of *T. gondii *in the studied population was higher than recorded by the Chinese Ministry of Health, and importantly, results suggest that women have a higher risk of being infected by *T. gondii *than men in China. The data argues for more attention for prevention of *T. gondii *infection in the female population and in particular pregnant women.

## Methods

### Study populations and Serum samples

2634 serum samples from clinically healthy individuals were collected in Changchun, Daqing and Shanghai areas (with a population of more than 100 million) from July 2006 to June 2008 (Table 1). 547 serum samples were collected from patients with psychiatric disorders (including Schizophrenia, mania, depression and severe stress) in Changchun regions (Table 1). The age of the studied population spanned from 15 to 65 yeas of age. The study was carried out with permission from the Ethical Committee of Institute of Zoonosis, Jilin University, China (Ref number 20070106). The sera were collected with agreement from the volunteers or patients. Written consensus was obtained from parents of all participating teenagers.

**Table 1:** Number of serum samples from study regions, gender distribution and seroprevalence.

	Clinically healthy persons (nr of positive sera)		P-value	Psychiatric patients (nr of positive sera)		P-value
**Region (Sample nr)**	**Female**	**Male**		**Female**	**Male**	
Changchun (1530)	536 (75)	447 (47)	0.1	319 (31)	228 (31)	
Daqing (758)	485 (64)	273 (23)	0.048	-	-	
Shanghai (893)	294 (49)	599 (70)	0.04	-	-	

Seroprevalence (%)	14	10.7	0.004	9.7	13.6	0.429

### Antigen

*T. gondii *tachyzoites (RH strain) were routinely maintained by cultivation in BHK (baby hamster kidney) cell lines as described earlier [16]. Briefly parasites from freshly lysed host cells were harvested, washed in PBS and disseminated by sonication. The cell debris was eliminated by centrifugation and the soluble antigens were collected and diluted to a final concentration of 1 mg/ml in PBS for the serological test.

### Serological assay

The *Toxoplasma*-specific IgG antibodies in 3181 serum samples were tested by Indirect Enzyme Linked Immunosorbant Assay (ELISA) [17] and the results were further verified by direct agglutination test with the Toxo-Screen DA (BioMerieux, France) kit. Briefly, Maxisor micro-ELISA plates (Nalge Nunc International, IL, USA) were coated with 50 μl per well of the *T. gondii *antigen (5 μg/ml) at 4°C overnight. 100 μl of each serum sample diluted at 1:50 was added to the wells in triplicates. Alkaline phosphatase-labeled goat anti-human IgG (Sigma, St. Louis, MO, 1:2000 dilution) and NPP [4-Nitrophenyl phosphate disodium salt hexahydrate] (Sigma, St. Louis, MO) were used to detect the antigen-antibody reaction. The plates were finally read in a Biotek 93 micro-ELISA auto reader 808 at 405 nm. Negative control wells with a negative serum verified by direct agglutination test were included in every plate. The cut-off point of OD values of a positive sample was set to be at least two times higher than that of the negative samples at any dilution point. Finally all sera with positive reactivity in ELISA were further tested with the commercial agglutination kit (Toxo-Screen DA, BioMérieux, France), according to the protocol provided by the manufacturer.

### Statistical Analysis

Results were analyzed with SPSS 15.0 software package. Chi square test was used to analyze the anti-*T. gondii *IgG seroprevalence in respect of gender, age, residence and psychiatric disorders of the populations. The differences were considered to be statistically significant when the P value was less than 0.05. Logistic regression analysis was used to assess the association with gender, age, residence and psychiatric disorders of the subjects and *T. gondii *infection in populations. Adjusted odds ratio (OR) and 95% confidence interval (CI) were calculated by multivariate analysis using logistic regression.

## Results

The overall prevalence of anti-*T. gondii *IgG in the studied population was 12.3%. In the clinically healthy group the prevalence was 12.5% meanwhile 11.3% in the group containing psychiatric patients (Table 1 and 2). No correlation between *T. gondii *infection and psychiatric disorders was found. A significant observation was the difference in seroprevalence between male and female groups where 10.5% of the males and 14.3% of the females were *T. gondii *positive (P = 0.004) (Table 1). The general seroprevalence of individuals living in Changchun, Daqing and Shanghai regions were 12.4%, 11.5%, and 13.3% respectively. Significant differences in prevalence between male and female were found in the populations of Daqing in the north and Shanghai in the south, where the prevalences were 8.4% versus 13.2% (P = 0.048) and 11.78% versus 16.7% (P = 0.04) respectively. The prevalence in Changchun was 10.5% and 14.0% respectively in men and women (P = 0.1).

**Table 2:** Number of psychiatric patients and seroprevalence.

Disorders	Patient number	Number of patients with *T. gondii *IgG and prevalence (%)
Schizophrenia	291	26 (8.9)
Mania	49	5 (10.2)
Depressive episode	29	4 (13.8)
Dissociative disorder	28	3 (10.7)
Severe stress and adjustment disorders	15	3 (20)
Obsessive-compulsive disorder	12	3 (25)
Other unspecified mental disorders	123	18 (14.6)

Total	547	62 (11.3)

Sera which had anti-*T. gondii *antibodies were divided into 5 groups based on the age (<20, 20-29, 30-39, 40-49 and >50 years) of the individuals. The seroprevalence of the five age groups were 11.2%, 11.8%, 12.6%, 13.6% and 12% respectively in the clinically healthy population. The seroprevalence in the corresponding age groups of the psychiatric patients were 9.1%, 12.8%, 7%, 13.1% and 12.9% respectively. The difference in infection rate among the sample groups was not statistically significant. However, when comparing the seroprevalence adjusted by age, the infection rates in women and men of the 20-30 years old group were 14.6% and 6.4% respectively. The difference was significant (P = 0.006). The prevalence of *T. gondii *infection in individuals living in urban and rural areas in the northern parts of China (including Changchun and Daqing) were 13.1% and 9.7%.

**Figure 1:**
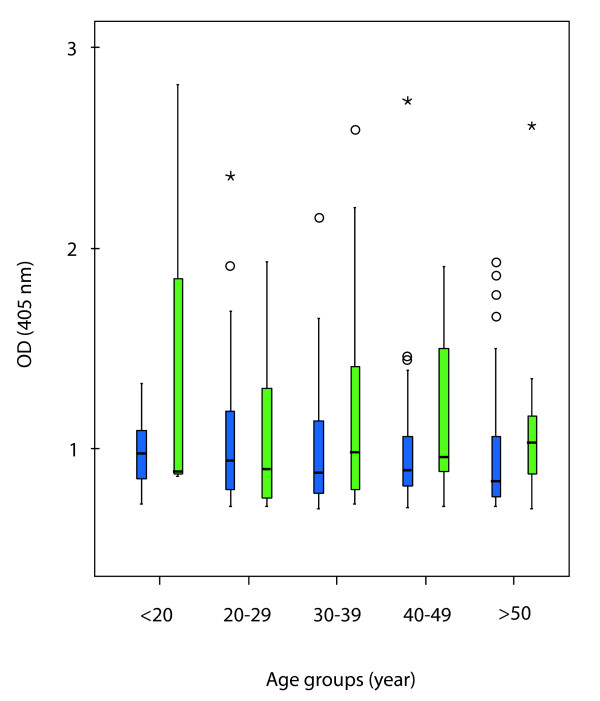
**Reactivities of antibodies to *T. gondii *antigen in the sera of different age groups**. The sera from clinically healthy group (blue) and psychiatric patients (green) were divided based on the age of each individual and the OD values from all samples in each group with specific reactivity were presented. Bars stand for 50% OD values of each group, lines below and above each bar stand for 25% OD values. The black lines in the bars stand for the median OD value. Samples with much higher or extreme OD values excluded in the statistical analysis are indicated with circles and asterisks.

The optical density at 405 nm (OD 405), indicated the amount of specific IgG antibodies in the sera, was also measured in the different samples. No significant differences were observed considering the mean values of all studied groups (Figure 1).

In the logistic regression analysis, ten variables (including age, living location, profession, physical condition and so on) were investigated for potential factors associated with *T. gondii *infection. The results revealed that the only factor associated with *T. gondii *infection was gender (OR = 0.712; 95% CI: 0.564-0.899). The rest of the factors did not show any association with *T. gondii *infection.

All positive and some negative samples investigated were verified by agglutination test using the commercially available Toxo-Screen DA kit (BioMérieux, France) as controls. No disagreement between ELISA and DA method was observed (data not shown).

## Discussion

Seroprevalence of *T. gondii *infection in humans and animals including chickens has been used as an indicator of the endemicity of the parasite [18-21]. The epidemiology of toxoplasmosis in many countries has been investigated, but the prevalence of *T. gondii *in the Chinese population has not been clear. During the two national-wide surveys carried out in 1995 and 2004 [22], the prevalence of *T. gondii *infection in the Chinese population was reported to be around 7%. But the number of samples enrolled in the two surveys was limited. In the current study, we investigated more than 3000 individuals living both in the south and the north of the country. Furthermore, the number of serum samples analyzed from male and female individuals was similar (Table 1). We also investigated the sera prevalence of *T. gondii*-specific IgG in more than 500 patients with psychiatric disorders, with the aim of finding a possible correlation between *T. gondii *infection and certain psychiatric symptoms as reported by others [8-15]. The overall infection rate of *T. gondii *in the studied population in China was 12.3%, which was generally lower than that in other countries [1,2]. Earlier studies suggest that the infection rate of free-range chickens in the northeast part of China were 34.5% [23]. Furthermore, it has recently been reported that cat infection rate of *T. gondii *in China is as high as 79.4% [24]. The low prevalence in humans found here could be due to the fact that the habit of consuming raw or undercooked meat and having pet animals still not very popular in China. No significant difference in infection rate between individuals living in urban and rural areas in the North part of China was found, which indicated that *T. gondii *infection is independent of living places. Thus consuming well-cooked meat and proper handling and disposal of faecal material from pet cats are important measures of disease prevention.

The finding that seroprevalance of anti-*T. gondii *IgG in the females is higher than in the males in the Chinese population is significantly important to the public health. The data indicates that females are at higher risk of contraction *T. gondii *infection in China. This is undoubtedly due to many factors. First, women traditionally take more care of pet animals including cats at home, and secondly, women handle raw meat more frequently than men due to the fact that they spend more time cooking at home. In a recent study with sera from pregnant women living in northeastern China, it was indeed found that taking care of pet animals and consuming raw meat were two main risk factors related to *T. gondii *infections [5]. The reason that individuals under the age of 20 showed similar infection rate to others could be due to the fact that they tend to play more with animals or due to other unidentified factors. Though it is rare that parasites are reactivated during gestation, infection during pregnancy does pose threat to the life of fetuses and newborns. Thus knowledge of disease prevention is more important in public health programs, especially to the female groups.

In immune competent individuals, *T. gondii *infects a variety of cells of the central nervous tissues, thus the correlation between *T. gondii *infections and certain psychiatric symptoms, especially Schizophrenia, has drawn attention in recent years [10-14]. It has been speculated that parasite components released from the infected brain cells might has effect on surrounding cells and hence impact the mode or behavior of the host [11]. Several studies including a literature surveys supported the conclusion that *T. gondii *infection is modestly associated with Schizophrenia syndrome [15]. However, no correlation between *T. gondii *infection and Schizophrenia was found in this study (n = 291, Table 2). Interestingly, the infection rate in the study group was only 8.9% which was lower than the average rate in the whole population. The patient groups with severe distress and adjustment disorders and Obsessive-compulsive disorder had serum positivity of 20% and 25% respectively. However, the figures are not conclusive due to the lower number of patients in the two groups. Unlike previous report, no correlation between *T. gondii *infection and psychiatric disorders were found in this study. This could be due to the genetic background of the Chinese population or the general low infection rate.

## Conclusions

The study has shown that the general infection rate of *T. gondii *in the Chinese population was 12.3%. Women have a significantly higher risk of being infected by *T. gondii *than men, possibly due to more exposure to the infective sources. No correlation between *T. gondii *infection and psychiatric disorders was observed.

## Competing interests

The authors declare that they have no competing interests.

## Authors' contributions

YX, JY, NJ, MX, LH, HL, HS, XL and HX participated in serum collection and performed the assay. JA, JL provided the antigen. QC planed and supervised the study. All authors contributed to the writing of the manuscript.

## Pre-publication history

The pre-publication history for this paper can be accessed here:

http://www.biomedcentral.com/1471-2334/10/4/prepub

## References

[B1] Tenter AM Heckeroth AR Weiss LM *Toxoplasma gondii*: from animals to humans Int J Parasitol 2000 30 1217 1258 10.1016/S0020-7519(00)00124-7 11113252 PMC3109627

[B2] Montoya JG Liesenfeld O Toxoplasmosis Lancet 2004 363 1965 1976 10.1016/S0140-6736(04)16412-X 15194258

[B3] Dubey JP The history of *Toxoplasma gondii*--the first 100 years J Eukaryot Microbiol 2008 55 467 475 10.1111/j.1550-7408.2008.00345.x 19120791

[B4] Alvarado-Esquivel C Torres-Castorena A Liesenfeld O García-López C Estrada-Martínez S Sifuentes-Álvarez A Marsal-Hernández J Esquivel-Cruz R Castañeda A Dubey JP Seroepidemiology of *Toxoplasma gondii *infection in pregnant women in rural Durango, Mexico J Parasitol 2009 95 271 274 10.1645/GE-1829.1 18922040

[B5] Liu Q Wei F Gao S Jiang L Lian H Yuan B Yuan Z Xia Z Liu B Xu X Zhu XQ *Toxoplasma gondii *infection in pregnant women in China Trans R Soc Trop Med Hyg 2009 103 162 166 10.1016/j.trstmh.2008.07.008 18822439

[B6] Nissapatorn V Lessons learned about opportunistic infections in Southeast Asia Southeast Asian J Trop Med Public Health 2008 39 625 641 19058599

[B7] Elsheikha EM Congenital toxoplasmosis: Priorities for further health promotion action Public Health 2008 122 335 353 10.1016/j.puhe.2007.08.009 17964621

[B8] Kravetz JD Federman DG Toxoplasmosis in pregnancy Am J Med 2005 118 212 216 10.1016/j.amjmed.2004.08.023 15745715

[B9] Walker M Zunt JR Parasitic central nervous system infections in immunocompromised hosts Clin Infect Dis 2005 40 1005 1015 10.1086/428621 15824993 PMC2692946

[B10] Torrey EF Yolken RH *Toxoplasma gondii *and schizophrenia Emerg Infect Dis 2003 11 1375 10.3201/eid0911.030143 PMC3035534 14725265

[B11] Carruthers VB Suzuki Y Effects of *Toxoplasma gondii *infection on the brain Schizophr Bull 2007 33 745 751 10.1093/schbul/sbm008 17322557 PMC2526127

[B12] Alvarado-Esquivel C Alanis-Quiñones OP Arreola-Valenzuela MA Rodríguez-Briones A Piedra-Nevarez LJ Duran-Morales E Estrada-Martínez S Martínez-García SA Liesenfeld O Seroepidemiology of *Toxoplasma gondii *infection in psychiatric inpatients in a northern Mexican city BMC Infect Dis 2006 6 178 10.1186/1471-2334-6-178 17178002 PMC1764421

[B13] Hinze-Selch D Däubener W Eggert L Erdag S Stoltenberg R Wilms S A controlled prospective study of *Toxoplasma gondii *infection in individuals with schizophrenia: beyond seroprevalence Schizophr Bull 2007 33 782 788 10.1093/schbul/sbm010 17387159 PMC2526145

[B14] Yolken RH Bachmann S Ruslanova I Lillehoj E Ford G Torrey EF Schroeder J Antibodies to *Toxoplasma gondii *in individuals with forst-episode schizophrenia Clin Infect Dis 2001 32 842 844 10.1086/319221 11229859

[B15] Torrey EF Bartko JJ Lun ZR Yolken RH Antibodies to *Toxoplasma gondii *in patients with schizophrenia: a meta-analysis Schizophr Bull 2007 33 729 736 10.1093/schbul/sbl050 17085743 PMC2526143

[B16] Lindström I Kaddu-Mulindwa DH Kironde F Lindh J Prevalence of latent and reactivated *Toxoplasma gondii *parasites in HIV-patients from Uganda Acta Trop 2006 100 218 222 10.1016/j.actatropica.2006.11.002 17157795

[B17] Balsari A Poli G Molina V Dovis M Petruzzelli E Boniolo A Rolleri E ELISA for *Toxoplasma *antibody detection: a comparison with other serodiagnostic tests J Clin Pathol 1980 33 640 643 10.1136/jcp.33.7.640 7430369 PMC1146176

[B18] Jones JL Kruszon-Moran D Wilson M Navin TR Gibbs R Schulkin J *Toxoplasma gondii *infection in the United States: seroprevalence and risk factors Am J Epidemiol 2001 154 357 365 10.1093/aje/154.4.357 11495859

[B19] Studenicová C Bencaiová G Holková R Seroprevalence of Toxoplasma gondii antibodies in a healthy population from Slovakia Eur J Int Med 2006 17 470 473 10.1016/j.ejim.2006.07.007 17098589

[B20] Sousa OE Saenz RE Frenkel JK Toxoplasmosis in Panama: a 10-year study Am J Trop Med Hyg 1988 38 315 322 3354766 10.4269/ajtmh.1988.38.315

[B21] Ades AE Parker S Gilbert R Tookey PA Berry T Hjelm M Wilcox AH Cubitt D Peckham CS Maternal prevalence of *Toxoplasma *antibody based on anonymous neonatal serosurvey: a geographical analysis Epidemiol Infect 1993 110 127 33 10.1017/S0950268800050755 8432316 PMC2271970

[B22] Xu LQ Chen YD Sun FH A national survey on current status of the important parasitic diseases in human population Chin J Parasitol Parasit Dis 2005 23 332 339 16562464

[B23] Zhu J Yin J Xiao Y Jiang N Ankarlev J Lindh J Chen Q A sero-epidemiological survey of *Toxoplasma gondii *infection in free-range and caged chickens in northeast China Vet Parasitol 2008 158 360 363 10.1016/j.vetpar.2008.09.024 19022581

[B24] Dubey JP Zhu XQ Sundar N Zhang H Kwok OC Su C Genetic and biologic characterization of *Toxoplasma gondii *isolates of cats from China Vet Parasitol 2007 145 352 356 10.1016/j.vetpar.2006.12.016 17267132

